# Self-reported prevalence of chronic non-communicable diseases and associated factors among older adults in South Africa

**DOI:** 10.3402/gha.v6i0.20936

**Published:** 2013-09-19

**Authors:** Nancy Phaswana-Mafuya, Karl Peltzer, Witness Chirinda, Alfred Musekiwa, Zamakayise Kose, Ebrahim Hoosain, Adlai Davids, Shandir Ramlagan

**Affiliations:** 1 HIV/AIDS/STI/and TB (HAST), Human Sciences Research Council, Pretoria, South Africa; 2 Office of the Deputy Vice Chancellor: Research and Engagement, Nelson Mandela Metropolitan University, Port Elizabeth, South Africa; 3 Department of Psychology, University of Limpopo, Turfloop Campus, Sovenga, South Africa; 4 Faculty of Health Sciences, Wits Reproductive Health and HIV Institute, University of the Witwatersrand, Johannesburg, South Africa

**Keywords:** self-reported, chronic non-communicable diseases, ageing, South Africa

## Abstract

**Introduction:**

Little is known about the prevalence and predictors of chronic non-communicable diseases (NCDs) of older adults in South Africa. This study aims to investigate the self-reported prevalences of major chronic NCDs and their predictors among older South Africans.

**Methods:**

We conducted a national population-based cross-sectional survey with a sample of 3,840 individuals aged 50 years or above in South Africa in 2008. The outcome variable was the self-reported presence of chronic NCDs suffered, namely, arthritis, stroke, angina, diabetes, chronic lung disease, asthma, depression, and hypertension. The exposure variables were sociodemographic characteristics: age, gender, education, wealth status, race, marital status, and residence. Multivariate logistic regression was used to determine sociodemographic factors predictive of the presence of chronic NCDs.

**Results:**

The prevalence of chronic NCDs was 51.8%. The prevalence of multimorbidity (≥2 chronic conditions) was 22.5%. Multivariate logistic regression analysis showed that being female, being in age groups 60–79 and 70–79, being Coloured or Asian, having no schooling, having greater wealth, and residing in an urban area were associated with the presence of NCDs.

**Conclusion:**

The rising burden of chronic NCDs affecting older people places a heavy burden on the healthcare system as a result of increased demand and access to healthcare services. Concerted effort is needed to develop strategies for the prevention and management of NCDs, especially among economically disadvantaged individuals who need these services the most.

Chronic non-communicable diseases (NCDs) are the principal cause of death; of the 57 million global deaths in 2008, 36 million (or 63%) were due to NCDs (1). Eighty percent (80%) of all of these deaths occur in low- and middle-income countries (1, 2). NCD deaths are projected to rise by 15% globally between 2010 and 2020. The greatest increases are projected to be in low- and middle-income regions like the African region, where they are projected to increase by more than 20% (1). The prevalence of NCDs is predicted to cause almost three-quarters as many deaths as communicable, maternal, perinatal, and nutritional diseases by 2020, and to exceed them as the most common causes of death by 2030 in Africa (2). The most common chronic NCDs reported globally include cardiovascular diseases, diabetes, cancer, and chronic respiratory diseases (3). A similar pattern has been observed in South Africa (4, 5). The impact of NCDs is far-reaching; because they threaten the economies of many countries, place high demands on a health service delivery system that is undergoing transformation in the face of shrinking budgets, and impact negatively on the health of older and experienced members of the workforce (because, as people age, their health deteriorates) (1, 6–8).

This is of greatest concern in South Africa given the fact that the size of the older population is rapidly increasing and is estimated to grow at a pace over four times the rate of the total population (9). Overall, the country has the second largest population aged 60 years or above in sub-Saharan Africa (10). Yet, little is known about the prevalence of chronic NCDs in the population aged 50 years and above in South Africa. It is critical to generate evidence on the magnitude of chronic NCDs among the elderly population not only to develop a national surveillance system but also to inform the development of strategies for the prevention of NCDs as well as to strengthen the healthcare system (5, 11). This study aims to investigate the prevalence and predictors of chronic NCDs among older South Africans who participated in the Study of Global Ageing and Adult Health (SAGE wave 1) in 2008.

## Methods

We conducted a national population-based cross-sectional survey with a sample of 3,840 individuals aged 50 years or above in South Africa in 2008. The SAGE sample design entails a two-stage probability sample that yields national estimates to an acceptable precision at provincial level, by locality type (urban and rural) and by race (including Black, Coloured, Asian, and White). The individual response rate among those aged 50 years or above was 77%. The SAGE wave 1 survey was carried out in South Africa by the Human Sciences Research Council (HSRC) in partnership with the World Health Organization (WHO) and the South African National Department of Health (NDOH). The study was approved by the HSRC Research Ethics Committee (Protocol REC 5/13/04/06) and the NDOH (J1/14/45, 2007).

The SAGE survey instruments and methods were adapted from those used by the World Health Survey (WHS) and were informed by a review of 16 surveys on ageing, including the US Health and Retirement Survey (HRS) and the English Longitudinal Study of Ageing (ELSA). The instruments assessed health status and health systems from a household and individual perspective. Standardized SAGE survey instruments were used in all countries and consisted of five main parts: (i) a household questionnaire; (ii) an individual questionnaire; (iii) a proxy questionnaire; (iv) a verbal autopsy questionnaire (VAQ); and (v) appendices, including showcards. The procedures for including country-specific adaptations to the standardized questionnaire and translations into local languages from English follow those developed by and used for the WHS. The questionnaire was interview administered. More detailed explanations of research methods for this study have been provided elsewhere (12).

### Measures

The outcome variable was the self-reported diagnosis of chronic NCDs which was previously made by a health professional; examples of such NCDs are arthritis, stroke, angina, diabetes, chronic lung disease, asthma, depression, and hypertension. These were assessed by self-reporting through answers to the question ‘Have you ever been diagnosed with diabetes (high blood sugar)?’ for example. The exposure variables were sociodemographic characteristics: age, gender, education, socioeconomic (wealth) status, race, marital status, and residence.

To estimate economic or wealth status, a random-effects probit model was used to identify indicator-specific thresholds that represent the point on the wealth scale above which a household was more likely to own a particular asset than not. This enabled an estimation of an asset ladder. These estimates of thresholds, combined with actual assets observed to be owned for any given household, were used to produce an estimate of household-level wealth status. This measure was used to create wealth tertiles (13).

A further question was asked regarding whether they had been taking any medications or other treatment for any of the aforementioned chronic NCDs in the past 12 months.

The data from the survey were captured on CSPro and analyzed using STATA Version 10. Data were weighted using post-stratified individual probability weights based on the selection of probability at each stage of selection. Individual weights were post-stratified by province, sex, and age groups according to the 2009 Medium Mid Year population estimates from Statistics South Africa (14). Multivariate logistic regression was used to determine sociodemographic factors predictive of the outcome – the presence of chronic NCDs (coded 1=yes, 0=no). In the analysis, weighted percentages have been reported. Both the reported 95% confidence intervals (CIs) and the *p*-value were adjusted for the multistage stratified cluster sample design of the study.

## Results

### Prevalence of self-reported chronic NCDs by sociodemographic factors

The prevalence of the eight chronic NCDs is shown in Table 1. The most prevalent chronic NCDs reported across the sample were hypertension (30.3%) and arthritis (24.7%). The prevalence of hypertension was higher among women (63.8%), African Blacks (71.8%), individuals with higher wealth (47.1), married individuals (47.7%), and urban residents (69.7%). The distribution of arthritis was similar to that of hypertension, being higher among women (66.6%), African Blacks (64.1%), those with higher wealth (47.3%), married individuals (43.6%), and those residing in urban areas (69.5%). Chronic lung infection and depression were the least reported NCDs (2.9%) in the study sample.

**Table 1:** Prevalence of self-reported NCDs among older South Africans

	Self-reported NCDs, *N* (%)							
	
Sociodemographics	Arthritis	Stroke	Angina	Diabetes	Chronic lung infection	Asthma	Depression	Hypertension
All	851 (24.7)	139 (4.0)	219 (5.2)	360 (9.2)	89 (2.9)	165 (4.9)	113 (2.9)	1,121 (30.3)
Gender								
Men	264 (33.4)	61 (45.9)	75 (32.5)	127 (33.0)	53 (64.2)	70 (46.4)	73 (54.2)	385 (36.2)
Women	587 (66.6)	78 (54.1)	144 (67.5)	232 (67.0)	36 (35.8)	95 (53.6)	40 (45.8)	736 (63.8)
Age								
50–59	334 (43.3)	57 (39.4)	93 (46.4)	123 (38.1)	36 (43.2)	76 (43.1)	64 (55.5)	421 (39.4)
60–69	316 (37.6)	40 (35.3)	78 (36.7)	129 (935.6)	33 (37.8)	62 (42.4)	33 (32.1)	409 (37.4)
70–79	146 (12.8)	29 (11.8)	37 (14.9)	89 (22.5)	16 (17.1)	20 (6.5)	12 (6.40	225 (17.8)
80 and above	55 (6.3)	134 (13.5)	11 (2.0)	18 (3.8)	4 (2.0)	7 (8.1)	4 (5.9)	66 (5.5)
Race								
African Black	387 (64.1)	57 (58.7)	88 (65.7)	149 (62.9)	49 (59.4)	76 (69.5)	36 (72.7)	575 (71.8)
White	52 (7.6)	11 (8.6)	19 (10.2)	23 (12.1)	9 (21.7)	5 (6.0)	13 (7.3)	76 (9.0)
Coloured	190 (21.8)	33 (29.6)	42 (17.2)	68 (14.9)	13 (16.3)	41 (20.7)	28 (14.5)	216 (14.8)
Asian or Indian	100 (6.5)	13 (3.1)	37 (6.8)	69 (10.0)	9 (2.7)	18 (3.8)	20 (5.6)	111 (4.5)
Wealth status								
Low wealth	271 (35.3)	47 (28.9)	53 (25.9)	67 (26.4)	33 (39.0)	66 (49.2)	24 (35.9)	322 (32.6)
Medium wealth	171 (17.5)	31 (21.7)	57 (22.3)	82 (17.6)	23 (16.5)	37 (17.6)	25 (17.1)	256 (20.3)
High wealth	405 (47.3)	59 (49.4)	109 (51.8)	208 (55.9)	33 (44.6)	62 (33.3)	64 (47.1)	537 (47.1)
Education								
No schooling	215 (35.7)	32 (39.4)	59 (39.6)	78 (27.9)	24 (36.9)	39 (34.2)	23 (33.0)	264 (33.0)
Less than 7 years	173 (32.5)	37 (32.7)	41 (26.8)	89 (34.3)	20 (29.8)	35 (39.0)	30 (37.7)	256 (32.3)
8–11 years	159 (28.9)	22 (19.1)	32 (23.1)	79 (31.7)	16 (31.7)	32 (25.1)	24 (22.1)	187 (30.2)
12 or more years	28 (2.9)	6 (8.8)	11 (10.5)	17 (31.7)	2 (1.6)	3 (1.7)	5 (97.1)	40 (4.5)
Marital status								
Single	115 (13.0)	13 (9.6)	22 (9.6)	37 (13.3)	10 (8.0)	25 (15.9)	12 (7.7)	153 (12.7)
Married	360 (43.6)	65 (56.5)	100 (54.7)	180 (49.6)	40 (59.8)	71 (49.0)	48 (57.7)	487 (47.7)
Cohabiting	18 (3.2)	4 (1.2)	9 (4.6)	3 (0.5)	6 (10.1)	10 (3.7)	3.0 (6.5)	55 (5.9)
Separated or divorced	56 (7.8)	13 (10)	12 (8.1)	16 (2.8)	8 (5.0)	11 (2.7)	7 (5.5)	65 (5.1)
Widowed	282 (32.4)	42 (22.7)	72 (23.0)	120 (33.8)	23 (16.3)	47 (28.6)	40 (22.5)	345 (28.7)
Geolocality								
Rural	221 (30.5)	38 (36.3)	59 (28.6)	62 (21.1)	31 (34.9)	43 (29.9)	18 (33.5)	288 (30.3)
Urban	630 (69.5)	100 (63.7)	160 (71.4)	297 (78.9)	58 (65.1)	122 (70.1)	95 (66.5)	833 (69.7)

## Associations between the number of chronic NCDs and sociodemographic characteristics

About half (48.7%) of the older people reported that they did not have any chronic NCDs, while about a third (28.8%) had one chronic NCD and 22.5% reported more than two chronic NCDs (see Table 2). In the study sample, the number of chronic NCDs differed significantly by gender, age, marital status, wealth status, race, and residence (*p*<0.001). The number of chronic NCDs did not differ significantly by level of education (*p*=0.187).

**Table 2:** Associations between the number of chronic NCDs and sociodemographic characteristics

	Number of NCDs			
	
	0	1	≥2	Chi-square *p*-value
Total	1,754 (48.7)	1,055 (28.8)	829 (22.5)	
Sex				
Female	899 (42.7)	634 (30.9)	559 (26.4)	<0.001
Male	855 (56.3)	421 (26.0)	270 (17.6)	
Age				
50–59	849 (54.2)	454 (28.6)	298 (17.2)	<0.001
60–69	517 (42.3)	350 (30.5)	306 (27.2)	
70–79	264 (42.3)	184 (28.0)	176 (29.8)	
80 and above	124 (51.0)	67 (22.9)	49 (26.1)	
Marital staus				
Never married	219 (48.6)	170 (33.2)	94 (18.2)	<0.001
Currently married	877 (52.2)	466 (25.8)	369 (21.9)	
Cohabiting	116 (57.3)	52 (30.0)	21 (12.7)	
Separated or divorced	103 (46.3)	66 (32.3)	48 (21.4)	
Widowed	404 (39.8)	282 (30.7)	285 (29.5)	
Education				
No schooling	338 (45.7)	239 (27.6)	206 (26.7)	0.187
Less than 7 years	331 (41.2)	216 (32.0)	191 (26.7)	
8–11 years	291 (47.1)	197 (32.1)	145 (20.8)	
12 or more years	85 (61.8)	44 (23.9)	29 (14.3)	
Wealth status				
Low	788 (54.3)	394 (28.9)	206 (16.8)	<0.001
Medium	328 (48.7)	200 (27.3)	190 (24.0)	
High	633 (43.5)	453 (29.0)	430 (27.5)	
Race				
African Black	1,034 (51.4)	572 (29.3)	362 (19.3)	<0.001
White	125 (52.9)	66 (20.6)	63 (26.5)	
Coloured	257 (32.2)	218 (36.1)	172 (31.7)	
Indian or Asian	108 (34.8)	65 (30.7)	113 (34.5)	
Geolocality				
Urban	1,065 (45.8)	734 (29.3)	636 (24.9)	<0.001
Rural	689 (54.1)	320 (27.7)	193 (18.2)	

## Sociodemographic predictors of chronic NCDs

Multivariate logistic regression analysis showed that being female, being in age groups 60–79 and 70–79, being separated or widowed, being Coloured or Asian, having no schooling, having greater wealth, and residing in an urban area were associated with the presence of NCDs (Table 3).

**Table 3:** Multivariate logistic regression analysis for the outcome – the presence of chronic NCDs

	Unadjusted odds ration (OR) (95% CI)	*p*	Adjusted OR (95% CI)	*p*
Gender				
Male	1.00		1.00	–
Female	1.64 (1.43–1.87)	<0.001	1.75 (1.44–2.11)	<0.001
Age				
50–59	1.00	–	1.00	–
60–69	1.43 (1.23–1.66)	<0.001	1.51 (1.24–1.85)	<0.001
70–79	1.54 (1.28–1.86)	<0.001	1.59 (1.22–2.07)	0.001
80 and over	1.06 (0.80–1.39)	0.69	1.51 (0.98–2.31)	0.06
Marital status				
Currently married	1.00	–	1.00	–
Single	1.26 (1.03–1.55)	0.02	1.27 (0.97–1.68)	0.09
Cohabiting	0.66 (0.49–0.90)	0.008	1.03 (0.66–1.63)	0.89
Separated or divorced	1.16 (0.88–1.54)	0.30	1.57 (1.09–2.26)	0.02
Widowed	1.47 (1.25–1.72)	<0.001	1.28 (1.02–1.62)	0.04
Education				
12 or more years	1.00	–	1.00	–
8–11 years	1.36 (0.96–1.93)	0.082	1.28 (0.88–1.86)	0.19
Less than 7 years	1.43 (1.01–2.02)	0.04	1.38 (0.94–2.04)	0.10
No schooling	1.53 (1.09–2.16)	0.02	1.66 (1.12–2.47)	0.01
Wealth status				
Low	1.00	–	1.00	–
Medium	1.56 (0.88–1.49)	<0.001	1.33 (1.04–1.71)	0.02
High	1.83 (1.58–2.12)	<0.001	1.63 (1.30–2.07)	<0.001
Race				
White	1.00	–	1.00	–
African Black	0.87 (0.67–1.13)	0.31	1.10 (0.79–1.53)	0.57
Coloured	1.47 (1.10–1.97)	0.01	1.50 (1.05–2.15)	0.03
Indian or Asian	1.60 (1.13–2.25)	0.007	1.59 (1.08–2.34)	0.02
Geolocality				
Rural	1.00	–	1.00	–
Urban	1.73 (1.50–1.99)	<0.001	1.54 (1.25–1.90)	<0.001

## Discussion

The study revealed that about 50% of the sample had one chronic NCD and that the most prevalent self-reported chronic NCDs were hypertension and arthritis. This supports the assertion that the magnitude of NCDs is high in low-resource settings (1, 2, 15). This is attributed not only to a sedentary lifestyle and poor dietary habits but also to the negative effects of globalization, rapid urbanization, and changing trends of population ageing (6). Of even greater concern is that the 2010 Global Burden of Disease report (16) projects an increase in the disease burden attributed to chronic NCDs.

The prevalence of multimorbidity (≥ 2 conditions) was 22.5%, which is comparable to that of the United States (about 26%) (17). Other studies in low- and middle-income countries (18) and in high-income countries have reported even higher prevalences of multimorbidity (19–21). A systematic review has also reported wide ranges in the prevalence of multimorbidity, especially in the older age groups (22). It should, however, be noted that the differences observed in multimorbidity between South Africa and other countries may not be comparable due to sociodemographic differences. Furthermore, it should be stated that the chronic comorbidities highlighted in this study were self-reported, and therefore possibilities of information bias that might have contributed to underreporting of the prevalences cannot be overlooked, especially because individuals tend to underreport poor health. Important to note is the fact that the elderly constitute a group with the potential for more health problems, higher health costs, and more complex healthcare needs.

Similar to other studies (6, 23–25), increasing age, being female, being separated or widowed, being Coloured or Asian, having no schooling, having greater wealth, and residing in an urban area were associated with the presence of chronic conditions. Interventions geared towards equitable health service delivery, like South Africa's National Health Insurance (26), should aim to reach for and achieve sustained benefits for the elderly with the above-mentioned characteristics as they are at higher risk for NCDs.

Caution needs to be exercised in interpreting these results. Notwithstanding, the discussion in this article highlights the need for a better understanding of the magnitude and underlying causes of ill health and morbidity among older people in sub-Saharan Africa. These findings have implications for the demand for healthcare services, health expenditure, and health budgets. This study strengthens the evidence base on the magnitude of NCDs among the elderly population. However, there is still a need to understand how these patterns are evolving over time, the implications of those changes for older people and their families, and patterns of healthcare use over time. Follow-up surveys are therefore needed to monitor trends and patterns over time. The cross-sectional nature of SAGE wave I does not provide these. Thus, SAGE surveys will be repeated 2–3 times in 5–10 years, and based on this, it is anticipated that policies and programmes will be further refined. South Africa, like other developing countries, needs to be prepared to address the escalating demands of chronic diseases. Every country, regardless of the level of its resources, has the potential to make improvements in preventing and controlling chronic disease (27, 28). Population ageing and older persons’ health, well-being, and protection are key issues facing contemporary society, and South Africa is no exception (29). This study confirms the need for effective control of NCDs among the elderly. South Africa is bound by legislation to prioritize NCD prevention and care for the elderly through provisions in its constitution and a myriad of laws with direct bearing on elderly care, including the Aged Persons Amendment Act of 1998, the Domestic Violence Act of 1998, the Housing Development Schemes Act for Retired Persons of 1988, and the Social Assistance Act of 2004. Apart from national laws and policies, South Africa is also a signatory to international agreements and declarations such as the Madrid International Plan of Action on Ageing, the United Nations Principles for Older Persons, the Valetta Declaration, the WHO Policy Framework on Active Ageing, and the African Union's Policy Framework and Plan of Action on Ageing. Future research will be imperative, and future waves of SAGE will be an ideal conduit for policy refinement, as well as support for the monitoring and evaluation of health programming for the elderly.

## Conclusion

The rising burden of chronic NCDs affecting older people places a heavy burden on the health system as a result of increased demand and access to healthcare services. Concerted effort is needed to develop strategies for the prevention and management of NCDs, especially among economically disadvantaged individuals who need these services the most.
